# Refractive Accuracy of a Novel Swept-Source OCT in Patients With Short and Long Eyes

**DOI:** 10.1155/joph/9987580

**Published:** 2025-01-20

**Authors:** Laureano A. Rementería-Capelo, Inés Contreras, Jorge L. García-Pérez, Javier Ruiz-Alcocer

**Affiliations:** ^1^Cornea and Refractive Surgery Unit, Clínica Rementería, Madrid, Spain; ^2^Instituto Ramón y Cajal de Investigaciones Sanitarias (IRYCIS), Hospital Universitario Ramón y Cajal, Madrid, Spain; ^3^Optics and Optometry Department, Universidad Complutense de Madrid, Madrid, Spain; ^4^Clinical and Experimental Eye Research Group, Universidad Complutense de Madrid, UCM 971009, Madrid, Spain

## Abstract

**Purpose:** To analyze the refractive accuracy of a novel swept-source optical coherence biometer (SS-OCT), that uses individual refractive indices to measure axial length, in short and long eyes implanted with monofocal intraocular lenses (IOLs).

**Methods:** This retrospective comparative study considered eyes with short axial length (AL) (< 22.5 mm) or long AL (> 26 mm) bilaterally implanted with the Acrysof IQ monofocal IOL. All eyes were preoperatively analyzed with the Argos biometer and IOL calculations were made using the Barrett Universal II (BUII). One month after the surgery, refractive and visual outcomes and refractive prediction errors were calculated. Furthermore, a back calculation of the prediction errors based on the Barrett True Axial Length (BTAL) formula was also performed and the results of both formulas were compared.

**Results:** Sixty eyes of 60 patients (30 with AL < 22.5 mm (short) and 30 with AL > 26 mm (long)) were included. After surgery, monocular UDVA was 0.03 ± 0.10 and 0.10 ± 0.15 logMAR for short-eye and long-eye groups, respectively. For short eyes, mean prediction error (MPE) with BUII and BTAL were 0.19 ± 0.34 D and 0.00 ± 0.35 D, respectively (*p*  <  0.001). Mean absolute error (MAE) was 0.32 ± 0.22 D with the BUII and 0.29 ± 0.20 D with the BTAL formula (*p*=0.21). For long eyes, MPE with BUII was −0.15 ± 0.35 D and −0.13 ± 0.36 D with BTAL (*p*=0.08), while MAE was 0.31 ± 0.21 D and 0.32 ± 0.20 D with BUII and BTAL, respectively (*p*=0.33). The percentage of eyes with a prediction error within ±0.5 D predicted postop spherical equivalent was > 75% for both groups and both formulas (*p* > 0.05 for all situations).

**Conclusions:** The novel SS-OCT biometer using individual refractive indices to measure AL showed an overall good refractive accuracy using the BUII. The results were similar or better with the optimized BTAL formula, suggesting that formulas purposely designed for biometric measurements with this novel technology are a promising tool for eyes with extreme AL.

## 1. Introduction

As an in vivo imagining tool, optical coherence tomography (OCT) has been one of the greatest advances in ophthalmology in the last decades. Its usefulness was initially related to retinal diseases detection, diagnosis, and monitoring. However, its properties have made it possible to expand the possibilities of image analysis, even allowing the prediction of visual function in patients through the analysis of retinal structure images [1]. In recent times, accessories have also been incorporated for a detailed study of the anterior pole of the eye and as with the posterior pole, its initial use was focused on visualization of structures. Even so, its use has become essential in the most performed surgery worldwide, which is cataract surgery [2], allowing for the calculation of intraocular lenses (IOLs).

In this field, conventional OCT biometers use the equivalent refractive index of each ocular tissue to calculate the axial length (AL) in patients with cataracts. However, novel swept-source OCT (SS-OCT) technology is available for clinicians, and it uses individual refractive indices to measure AL [3, 4]. With this technology, the AL is calculated by converting the optical distances of each ocular tissue into physical distances using individual refractive indices [3, 5]. The biometry results are crucial for calculating the IOL power and then achieving good postoperative refractive results. Different authors have reported satisfactory predictive accuracy in normal eyes measured with this novel technology [6, 7]; however, less is known related to accuracy of this device in short and long eyes.

In addition, different formulas can be used for IOL calculation, and this may be a key issue when considering eyes with nonconventional AL values [8]. Therefore, the aim of this study is to analyze the refractive accuracy of a novel SS-OCT biometer for patients that underwent cataract surgeries and monofocal IOL implantation with AL < 22.5 mm (short) and with AL > 26 mm (long). The IOL power for each patient was calculated with the Barrett Universal II (BUII) formula. Refractive accuracy based on back calculations with the Barrett True Axial Length (BTAL) formula was also ,analyzed and compared.

## 2. Methods

This retrospective comparative study was performed at Clínica Rementería, Madrid, Spain, and included patients who had undergone bilateral implantation of a monofocal IOL. The study followed the tenets of the Declaration of Helsinki and was reviewed and approved by the Ethics Committee of the Hospital Clínico San Carlos, Madrid. Informed consent was obtained from all patients prior to inclusion.

The study reviewed patients > 40 years old that underwent routine cataract surgery and monofocal nontoric IOL implantation without complications and with a final uncorrected distance visual acuity (UDVA) of 20/40 or better. The IOL implanted in all patients was the AcrySof IQ SN60WF (Alcon Laboratories, Inc, Fort Worth, USA). Patients with ocular comorbidities (glaucoma, severe dry eye, diabetic retinopathy, uveitis, retinal detachment, or prior refractive surgery), systemic diseases, or systemic medications that could affect the VA or the refraction of the patients, posterior capsule opacification and/or YAG laser capsulotomy were excluded for the study. Before each surgery, inclusion and exclusion criteria were assessed by an ophthalmologic examination including refraction, screening for ocular conditions and/or systemic diseases, biomicroscopy, and fundus examination. At the same time, the Pentacam Nucleus Staging (PNS) cataract grading score (from 0 to 5) was also assessed for all patients with the Pentacam (Oculus Inc., Germany).

For the analysis, patients were divided into two groups, one with short eyes (AL ≤ 22.5 mm) and the other with long eyes (AL ≥ 26 mm). One eye of each patient was analyzed to prevent the double organ bias [9].

### 2.1. Preoperative Clinical Assessment

Biometry and IOL calculations were performed with the Argos (Alcon Laboratories, Inc, Fort Worth, USA) SS-OCT device and the BUII formula. For all cases, the refractive target was “emmetropia.” As described by previous authors, the Argos biometer uses a 1060 nm wavelength and a 20 nm bandwidth swept-source technology to collect 2-dimensional OCT data of the eye [10, 11]. It uses a segmental refractive index for each segmental length (cornea, anterior chamber, lens, and vitreous) when calculating the AL. Then, the AL calculation is adjusted when there are variations in the relative lengths of these segments [10, 11].

### 2.2. Postoperative Clinical Assessment and Data Analysis

Patients followed 1-day, 1-week, and 1-month visits after the surgery and the results of the last visit were considered for the study. The mean (arithmetic) prediction error (ME), mean absolute prediction error (MAE), median absolute prediction error (MedAE), and percentage of eyes with a prediction error within ±0.25, ±0.5, ± 0.75, and ±1.00 D of predicted postop spherical equivalent were obtained based on surgical calculations with the BUII formula. A back calculation of the same parameters based on the BTAL formula was also performed and results were compared to those obtained with the BUII formula. The BTAL formula has been recently described as an updated version of the BUII formula that is designed for measurements that use the sum-of-segment methodology [8, 11] which is the case of the Argos biometer.

### 2.3. Statistical Analysis

All study data will be collected in an Excel database (Microsoft Office 2010; Microsoft). Data analysis was performed using SPSS for Windows V.20.0 (SPSS Inc, Chicago, USA). The normal distribution of variables was assessed using the Kolmogorov–Smirnov test. The *t*-test was used for comparing the ME and MAE. The chi-square test was employed to assess the percentage of eyes within different targets. Differences were considered to be statistically significant when the *p* value was < 0.05 (i.e., at the 5% level).

## 3. Results

Sixty eyes of 60 patients (30 with AL < 22.5 mm (short) and 30 with AL > 26 mm (long)) were included in the study and preoperative demographics of the sample are presented in Table 1. No adverse events were reported during the study.

**Figure 1:**
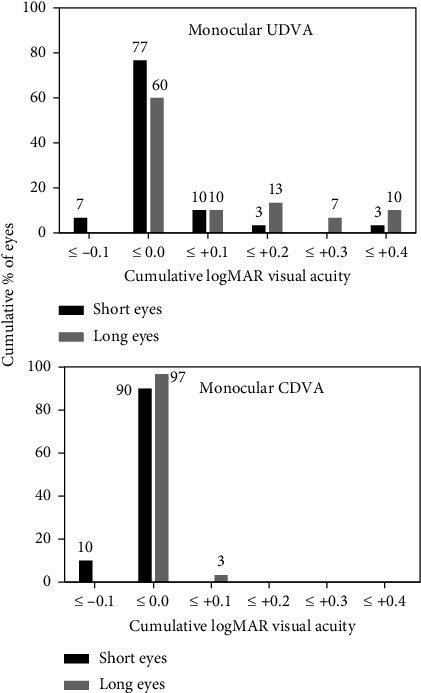
Distribution of monocular uncorrected and corrected distance visual acuity (UDVA and CDVA) for both groups 1 month after the surgery.

Mean monocular UDVA 1 month after the surgery was 0.03 ± 0.10 and 0.10 ± 0.15 logMAR for short-eye and long-eye groups, respectively. On the other hand, mean monocular CDVA was 0.00 ± 0.03 logMAR and 0.01 ± 0.02 logMAR for short and long eyes, respectively. The distributions of monocular UDVA and CDVA for both groups are presented in Figure 1.

**Figure 2:**
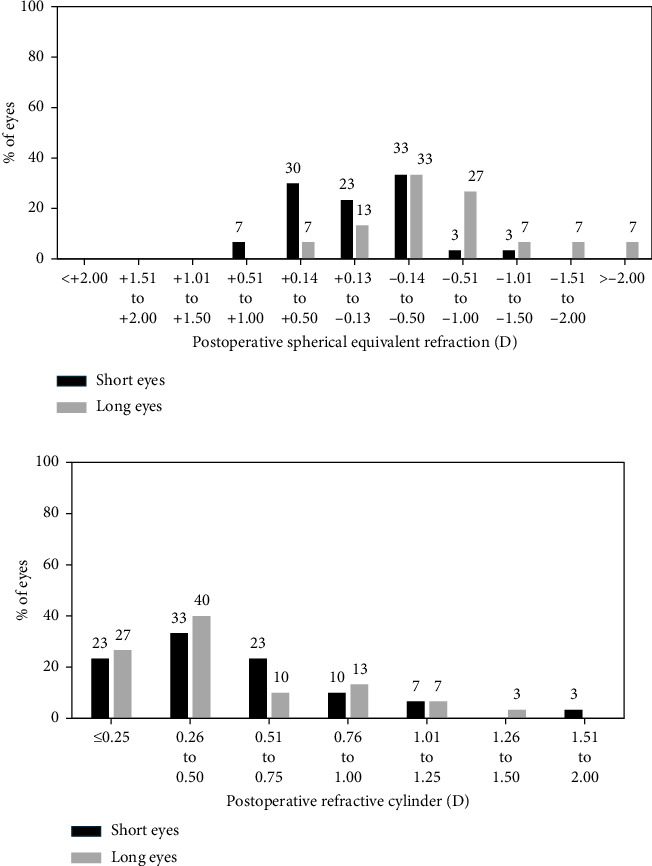
Postoperative refractive outcomes using the Barrett universal II (BUII) formula.

The mean spherical equivalent was −0.03 ± 0.47 D for the short eyes group and −0.67 ± 0.73 D for the long eyes group (*p*  <  0.001). Median cylinder was −0.5D (range: 0–−2 D) for the short eyes group and −0.5 D (range: −0.25–−1.5 D) for the long eyes group. Postoperative refractive outcomes using the BUII formula are shown in Figure 2.

**Figure 3:**
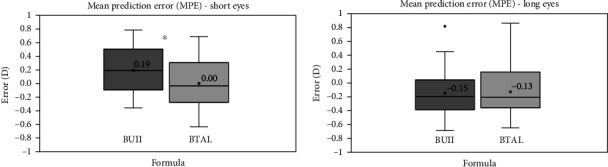
Mean prediction error (MPE) for short and long eyes groups with both the Barrett universal II (BUII) and the Barrett true axial length (BTAL) formulas.

MPE for short eyes with the BUII and the BTAL were 0.19 ± 0.34 D and 0.00 ± 0.35 D, respectively, showing statistically significant differences between both formulas (*p*  <  0.001). For the case of long eyes, MPE with the BUII formula was −0.15 ± 0.35 D and −0.13 ± 0.36 D with the BTAL. In this case, no statistically significant differences were found between both formulas (*p*=0.08). A box and whisker plot represents the MPE results for both groups in Figure 3.

**Figure 4:**
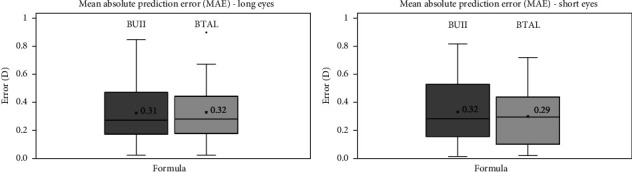
Mean absolute error (MAE) for short and long eyes groups with both the Barrett universal II (BUII) and the Barrett true axial length (BTAL) formulas.

MAE for the short eyes group was 0.32 ± 0.22 D with the BUII and 0.29 ± 0.20 D with the BTAL formula, showing no statistically significant differences (*p*=0.21). For the long eyes group, MAE was 0.31 ± 0.21 D and 0.32 ± 0.20 D with the BUII and the BTAL, respectively, showing no statistically significant differences between both formulas (*p*=0.33). Similarly, a box and whisker plot represent MAE results for both groups in Figure 4.

MedAE for short eyes with the BUII was 0.27 and 0.28 with the BTAL. For long eyes, MedAE was 0.28 and 0.27 with the BUII and the BTAL formula, respectively.

Finally, the percentage of eyes with a prediction error within ±0.25, ±0.5, ±0.75, and ±1.00 D for both groups is shown in Table 2. The Chi-square test found no differences in the distribution of residual errors (±0.25/±0.50/±0.75/±1.00) between the two formulas, neither for short eyes (*p*=0.62) nor for long eyes (*p*=0.99).

## 4. Discussion

Accuracy of AL measurements and IOL power calculations have been and are the key issues for achieving good refractive results after cataract surgery. The novel SS-OCT technology calculates the AL by converting the optical distances of each ocular tissue into physical distances using individual refractive indices of each segment of the eye [3, 11], adjusting potential variations in the relative lengths of these components to guarantee an appropriate AL calculation [8]. It has been suggested that using segmental refractive indices for each segment is more precise than the one using a single equivalent refractive index when considering eyes with nonstandard anatomies, for example, for short and long eyes, due to a single equivalent refractive index may overestimate or underestimate AL values, respectively [11]. To maximize the benefits of this accurate AL calculation, novel formulas should consider this technical approach. This issue gains importance in eyes with nonconventional AL values and this is why in the current study, we analyzed the refractive accuracy of a novel SS-OCT biometer with the classical BUII formula in short and long eyes. The results were then compared with those obtained with a back calculation performed with the BTAL formula, which is designed for measurements that use the sum-of-segment methodology.

Our clinical results 1 month after the surgery using the Argos biometer and the BUII formula show that UDVA was slightly better for short eyes, being the VA satisfactory for both groups (Figure 1). Mean refractive spherical equivalent of the analyzed sample was slightly negative for long eyes while short eyes achieved a mean spherical equivalent close to zero. In addition, observing Figure 2, it is possible to see that, despite the differences being minimal and postoperative refraction achieved almost the emmetropia, long eyes show a distribution of postoperative spherical equivalent that slightly trends toward negative refractive values if compared with short eyes. In a recent work in which the same SS-OCT device (Argos) and the BUII formula were used to calculated IOL power, Blehm and Hall achieved similar refractive outcomes, that is, postoperative refraction was slightly more negative for long eyes if compared with short eyes [12]. Nevertheless, in this previous work, patients were implanted with Extended Depth of Focus (EDoF) IOLs, and direct comparisons should be made with caution.

Despite the slight differences between short and long eyes, the refractive results with the BUII formula could be considered satisfactory. However, it would be interesting to analyze whether direct IOL calculations with SS-OCT biometers and formulas designed for the measurements that use the sum-of-segment methodology (such as BTAL) could offer even better refractive results in patients with short and long eyes.

When analyzing the accuracy of the formulas for both groups, the results showed certain variations. Observing Figure 3 (left part of the figure), it is possible to observe that arithmetic prediction error (MPE) for short eyes was significantly more positive with the BUII if compared with the BTAL for which the MPE was almost zero (0.19 ± 0.34 D with BUII vs. 0.00 ± 0.35 D with BTAL). These results agree with those reported by Shammas et al. [8], in which MPE for short eyes with the BUII was 0.09 ± 0.42 D and −0.07 ± 0.40 D with the BTAL. For long eyes, Figure 3 (right part of the figure) shows that MPE was slightly negative with both formulas showing no difference between them (−0.15 ± 0.35 D with BUII and −0.13 ± 0.36 D with BTAL). These results also agree with those obtained by Shammas et al. [8], in which MPE for long eyes was −0.08 ± 0.38 D and −0.06 ± 0.36 D with the BULL and BTAL, respectively.

On the other hand, observing absolute prediction error (MAE) results in Figure 4, it is possible to observe that, in both groups, both formulas showed similar prediction errors. The MAE for all cases, that is, for both groups and with the two formulas, showed to be around 0.30 D. Besides slight differences, it could be said the results agree with previous studies of Shammas et al. [8] and Blehm and Hall [12] and confirm the accuracy results for absolute refractive values of the SS-OCT biometer with these two formulas when considering short and long eyes. It should be mentioned that, in the study of Shammas et al., the authors described the accuracy of different formulas in short and long eyes, but they did not perform a direct statistical comparison for MPE and MAE values between BUII and BTAL formulas. In our study, we also found that in terms of MAE, both formulas perform similarly for both groups, however, if the sign of the error is considered (MPE), BTAL showed better results for the group of short eyes. This issue should be considered due to absolute errors can mask differences if a formula systematically fails toward negative values by a certain amount or if another formula fails sometimes toward positive and other times towards negative. If signs are considered, differences between formulas could be more evident, while the accuracy may be similar if absolute values are considered.

Finally, for short and long eyes, the proportion of eyes within ±0.25, ±0.5, ±0.75, and ±1.00 D showed no differences between both formulas. For short eyes, the proportion of eyes within ±0.25 D with both formulas was around 40%, around 80% within ±0.50 D and around 100% within ±0.75 and ± 1.00 D. For the case of long eyes, the results were similar and the proportion of eyes within ±0.25, ±0.50, ±0.75, and ± 1.00 D with both formulas were around 50%, 80%, 100%, and 100%, respectively. These results agree with those obtained by Shammas et al. [8] and were slightly worse for the values within ±0.25 if compared with those achieved by Blehm and Hall [12]. The slight differences may be due to differences in the samples employed; the IOLs employed in both studies and the higher variability for refractions under 0.25 D. Nevertheless, our results also confirm the good refractive accuracy of the Argos biometer with the BUII and BTAL formulas. Other authors reported more variability in the accuracy of this biometer in eyes with nonconventional AL values, achieving similar results for long eyes but slightly worse results in the proportion of eyes within ±0.50 D for short eyes (around 71%) [13]. To stress the relation between novel devices and new generation formulas, these authors employed the Haigis formula and direct comparisons should not be made.

It should be noted that cutoffs for long and short eyes are controversial and that the limits for these cutoffs may imply a significant difference for small sample sizes. Our results and the results of previous authors reported good refractive outcomes with these two formulas. However, the slight variability between both formulas in short eyes may show a trend toward higher differences if longer groups of extreme eyes (both short and long) are considered. Therefore, future studies should analyze the accuracy of these novel SS-OCT devices and IOL calculation with updated formulas designed for the measurements with biometers that use the sum-of-segment methodology.

Finally, unlike the studies cited in this discussion, in the current study, the grade of lens opacity was measured. Our sample showed an overall low grade of lens opacity (PNS = 1.7 ± 0.7 for short eyes and PNS = 1.2 ± 0.7 for long eyes). Previous studies reported that SS-OCT biometers have a low rate of failure when measuring AL regardless the severity of the cataract [14, 15]. However, other authors reported that the failure rate of AL measurements in conventional eyes with dense cataracts of eyes may achieve rates up to 20% when SS-OCT-based biometry was employed [16]. Besides the good results achieved with SS-OCT devices, clinicians should pay special attention to analyze whether high degrees of lens opacities combined with extremely short or long eyes may have an impact on SS-OCT measurements leading to errors on IOL power calculation.

It is important to note that the patients in our study were healthy and presented normal corneal profiles. However, in the last decades, a significant proportion of the population has undergone corneal refractive procedures. In this regard, myopic and hyperopic ablations induce remarkable changes on corneal topography [17]. Since these changes would have an impact on IOL calculations, future similar studies should also consider patients with previous corneal refractive surgeries. This may increase the potential of these novel devices and the related formulas.

In conclusion, it could be said that the Argos biometer showed an overall good refractive accuracy in short and long eyes implanted with monofocal IOLs using both the BUII and BTAL formula. In both groups, > 75% of the patients obtained a prediction error within ±0.50 D. However, the slight better results of prediction error obtained with the updated BTAL formula in short eyes should open a line of work to conclude if the refractive accuracy in longer groups with extreme AL may show differences if formulas designed for measurements that use the sum-of-segment methodology are considered.

## Figures and Tables

**Table 1:** Preoperative demographics.

	Age (years)	Pupil size (mm)	Axial length (mm)	PNS	IOL power (D)	AutoRx sphere (D)	AutoRx cylinder (D)	AutoRx spherical equivalent (D)
Short eyes AL < 22.5 mm	73.8 ± 6.9	4.3 ± 0.8	22.2 ± 0.3	1.7 ± 0.7	24.3 ± 1.8	2.4 ± 2.0	−0.8 ± 0.6	1.9 ± 2.0
Long eyes AL > 26 mm	69.5 ± 6.8	4.3 ± 1.2	27.1 ± 0.9	1.2 ± 0.7	10.8 ± 2.8	−8.4 ± 4.3	−1.0 ± 0.6	−8.7 ± 4.5

*Note:* Values are shown as the mean and standard deviation.

Abbreviations: AutoRx, automated refraction; IOLs, intraocular lens; PNS, Pentacam Nuclear Staging.

**Table 2:** Prediction error with both formulas and for both groups.

		Barrett universal II	Barrett true axial length
AL group	Prediction error (D)	Proportion of patients (%)	
Short eyes AL < 22.5 mm	**0.25**	40	43.3
	**0.50**	76.7	83.3
	**0.75**	96.7	100
	**1.0**	100	100

Long eyes AL > 26 mm	**0.25**	50	46.7
	**0.50**	80	80
	**0.75**	96.7	96.7
	**1.0**	100	100

*Note:* Values are shown as the proportion of patients (%) within ±0.25, ±0.50, ±0.75, and ±1.00 D of the predicted refraction. The bold values represent the prediction error in 0.25D steps from 0.25 to 1D. The rest of the values represent the proportion of patients in the mentioned ranges.

Abbreviation: AL, axial length.

## Data Availability

The datasets generated and/or analyzed during the current study are not publicly available but are available from the corresponding author upon reasonable request.
